# Dual-Layer Spectral CT for Advanced Tissue Characterization: Differentiating Bladder Neoplasm from Intraluminal Thrombus—A Case Report

**DOI:** 10.3390/reports8030186

**Published:** 2025-09-20

**Authors:** Bianca Catalano, Damiano Caruso, Giuseppe Tremamunno

**Affiliations:** 1 Faculty of Medicine and Psychology, Sant’Andrea University Hospital, Sapienza-University of Rome, 00189 Rome, Italy; bianca.catalano00@gmail.com; 2 Department of Medical Surgical Sciences and Translational Medicine, Radiology Unit-Sant’Andrea University Hospital, Sapienza-University of Rome, 00189 Rome, Italy; damiano.caruso@uniroma1.it

**Keywords:** bladder neoplasm, electron density, iodine density, spectral CT, thrombus, Z-effective

## Abstract

**Background and Clinical Significance:** Bladder neoplasms often present with coexisting thrombi and hematuria, appearing as complex intraluminal masses on imaging, and posing a key diagnostic challenge in distinguishing neoplastic tissue from thrombus, to prevent harmful overstaging. **Case Presentation:** An 82-year-old man with recurrent gross hematuria and urinary disturbances was evaluated by ultrasound, which identified a large endoluminal lesion in the anterior bladder wall. The patient subsequently underwent contrast-enhanced CT using a second-generation dual-layer spectral CT system, which utilizes a dual-layer detector to simultaneously acquire high- and low-energy X-ray data. Conventional CT images confirmed a multifocal, bulky hyperdense lesion along the bladder wall, protruding into the lumen and raising suspicion for a heterogeneous mass, though further characterization was not possible. Spectral imaging enabled the reconstruction of additional maps—such as iodine density, effective atomic number (Z-effective), and electron density—which were used to further characterize these findings. The combination of these techniques clearly demonstrated differences in iodine uptake and tissue composition within the parietal lesions, allowing for a reliable differentiation between neoplastic tissue and intraluminal thrombus. **Conclusions:** The integration of conventional CT imaging with spectral-derived maps generated in post-processing allowed for accurate and reliable tissue differentiation between bladder neoplasm and thrombus. Spectral imaging holds the potential to prevent tumor overstaging, thereby supporting more appropriate clinical management. The dual-layer technology enables the generation of these maps from every acquisition without altering the scan protocol, thereby having minimal impact on the daily clinical workflow.

## 1. Introduction and Clinical Significance

Bladder neoplasms represent the second most frequent malignancy of the genitourinary tract, following prostate cancer [1]. Their etiology is multifactorial, involving several risk factors: oncogenes, tumor suppressor genes, environmental exposures, and hereditary components. Initial diagnostic work-up should include a detailed medical history and a comprehensive physical examination. In most cases, painless macroscopic hematuria is the sole presenting symptom of the primary urothelial bladder cancer. Intraluminal thrombi are commonly observed in association with bladder neoplasms, representing a characteristic but often overlooked finding [2].

Although bladder neoplasms are ultimately diagnosed by cystoscopy with histological confirmation, CT urography is widely regarded as the first-line imaging modality in patients with macroscopic hematuria, providing a comprehensive evaluation of both bladder and upper urinary tract. In many cases, this approach is already diagnostic. In the presence of a bladder mass, the lesion typically appears as a hyperechoic vegetation in the bladder lumen, sometimes exhibiting a Doppler signal if the lesion is vascularized at the pedicle. Intraluminal thrombi, when present, appear as hyperechoic areas compared to the surrounding urine and can be challenging to characterize, especially when adherent to the bladder wall and to differentiate from the tumor.

Similarly to an abdominal ultrasound, urine cytology represents a valuable adjunct in the evaluation of bladder cancer, even though a negative cytology result does not exclude the presence of a low-grade carcinoma, while a positive result is more commonly associated with high-grade tumors [3].

Flexible cystourethroscopy, as a third-level diagnostic tool, allows direct visualization of the bladder mucosa, enabling tumor detection and targeted biopsy for definitive histological diagnosis [4].

In daily clinical practice, beyond ultrasound, the most widely used imaging modality for comprehensive evaluation and staging of the entire urinary tract is contrast-enhanced computed tomography urography (CT urography) [5]. This second-line investigation provides detailed visualization not only of the bladder and perivescical structures but also of the upper urinary tract, offering functional insights into renal parenchyma, lymph node volume, and potential non-neoplastic causes of hematuria, whether intrinsic or extrinsic.

In recent years, the introduction of spectral CT has enabled advancements beyond conventional imaging. Dual-energy CT (DECT) is based on the acquisition or detection of two distinct photon spectra, one at high energy and one at low energy, by exploiting the energy-dependent attenuation properties of tissues [6]. This approach primarily exploits differences in the photoelectric effect and Compton scattering to differentiate various materials. This technology allows the generation of advanced reconstructions, including iodine density maps and other compositional images, such as effective atomic number (Z-effective) and electron density maps, thereby enhancing tissue characterization [7].

Among the various DECT approaches, dual-layer spectral detector CT enables simultaneous acquisition of low- and high-energy data without the need to preselect a spectral protocol, making it well-suited for routine clinical practice [8].

This case report illustrates the potential benefits and clinical applications of the novel dual-layer DECT technology, particularly in the differentiation between a neoplastic bladder lesion and an adjacent intraluminal clot.

## 2. Case Presentation

An 82-year-old male presented to the Emergency Department with urinary symptoms and recurrent episodes of gross hematuria. His medical history was notable for remote tobacco use, with no prior urological conditions and no known family history of bladder cancer. No hematological disorders and specific risk factors for thrombus formation, such as anticoagulant therapy, recent surgery or prolonged immobilization, were identified. To further evaluate the clinical presentation, a suprapubic ultrasound was performed, revealing a large endoluminal mass apparently arising from the anterior bladder wall. This case was managed in accordance with the Declaration of Helsinki, and informed consent for participation was obtained from the patient [9].

### 2.1. Investigations

To further investigate, a contrast-enhanced CT examination (CT urography) was performed using a second-generation dual-layer detector-based spectral CT (CT 7500, Philips Healthcare, Best, The Netherlands). A total of 110 mL of non-ionic, high-iodine concentration contrast medium (400 mgI/mL iomeprol, Iomeron 400; Bracco Imaging, Milan, Italy) was intravenously injected at a fixed flow rate of 3 mL/s through an 18-gauge antecubital access, followed by a 40 mL saline chaser bolus administered at the same flow rate. The CT acquisition protocol included the late arterial phase, portal venous phase, and delayed phase.

### 2.2. Conventional CT Findings

**Figure 1:**
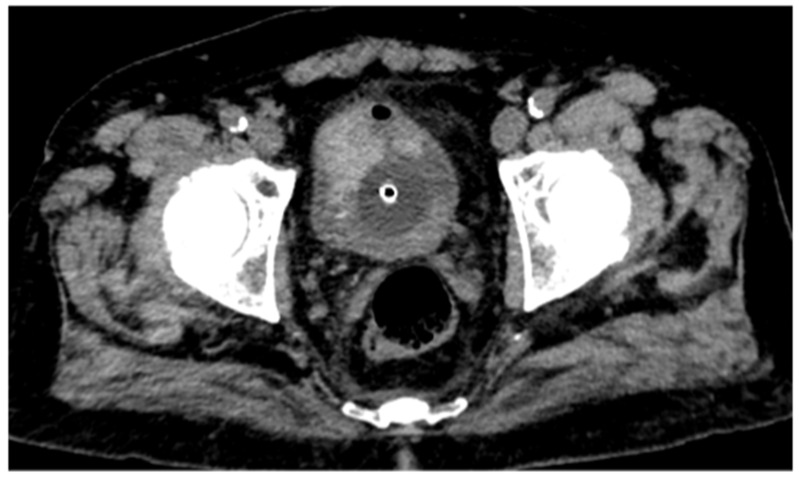
Conventional unenhanced CT scan shows a large hyperdense lesion on the anterior bladder wall and a mildly hyperdense area along the left posterolateral wall. Further evaluation with intravenous contrast medium administration is required to better characterize these findings.

CT urography, through its multiphase protocol, enables the identification of key radiological findings that contribute to diagnostic assessment. In the conventional unenhanced phase, two hyperdense areas are observed: one along the left posterolateral bladder wall, and another protruding into the lumen at the level of the anterior wall and bladder dome (Figure 1).

**Figure 2:**
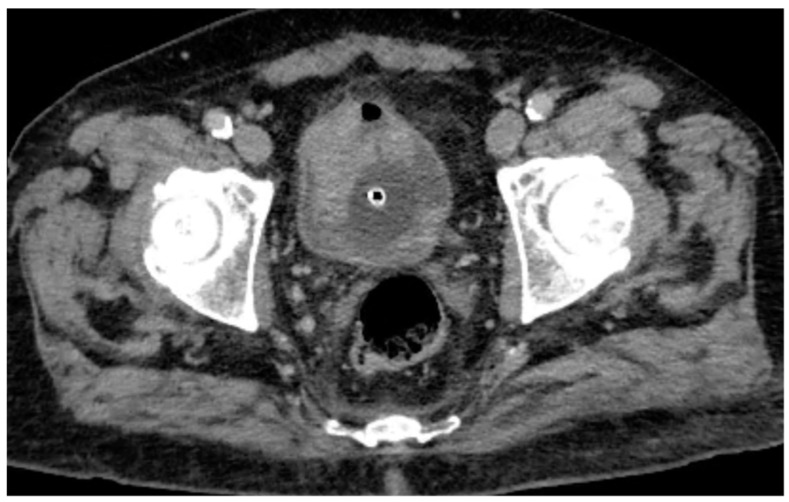
Venous phase CT scan confirms the previously described findings, showing a lesion involving the anterior and left posterolateral bladder wall, with an associated hyperdense intraluminal component.

Following intravenous administration of iodinated contrast medium, the venous phase confirms the presence of the hyperdense lesions along the anterior and left posterolateral bladder walls, with potential contrast enhancement that cannot be unequivocally quantified (Figure 2). Consequently, conventional CT imaging alone was insufficient to definitively characterize the nature of these findings.

### 2.3. Spectral CT Analysis

Dual-layer spectral CT technology allows for the post-processing of multiple maps that enhance lesion characterization. The iodine density map, which provides a color-coded representation of iodine concentration in mg/mL, enables quantitative assessment of contrast uptake within lesions.

**Figure 3:**
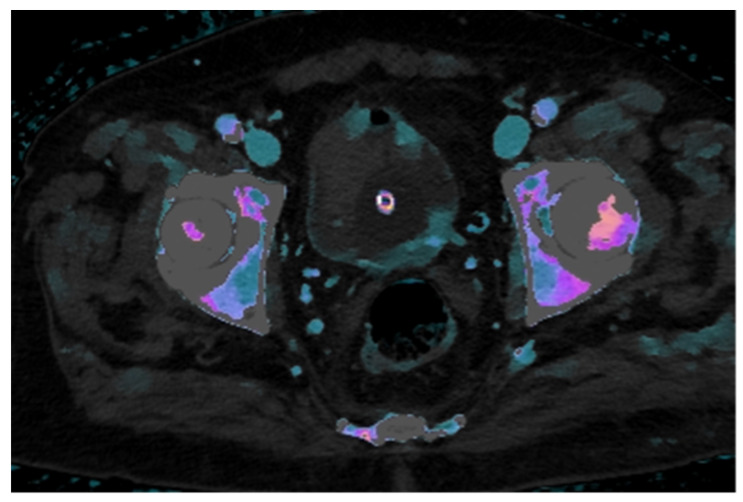
The iodine density map overlaid on venous phase CT images shows increased iodine uptake in the posterolateral left bladder wall and in two foci of the anterior wall, findings indicative of metabolically active neoplastic areas. In contrast, the mass in the right anterolateral wall demonstrates no iodine uptake, consistent with a non-metabolically active lesion, suggestive of thrombus.

In this case, the iodine map overlaid on the conventional venous phase image demonstrates clear increased iodine uptake in two foci along the left posterolateral bladder wall and in two minute foci along the anterior wall, findings consistent with hypervascular neoplastic tissue. In contrast, the intraluminal hyperdense mass, visible on the unenhanced scan, shows no significant iodine uptake, supporting the diagnosis of a non-enhancing structure, most likely a thrombus (Figure 3).

**Figure 4:**
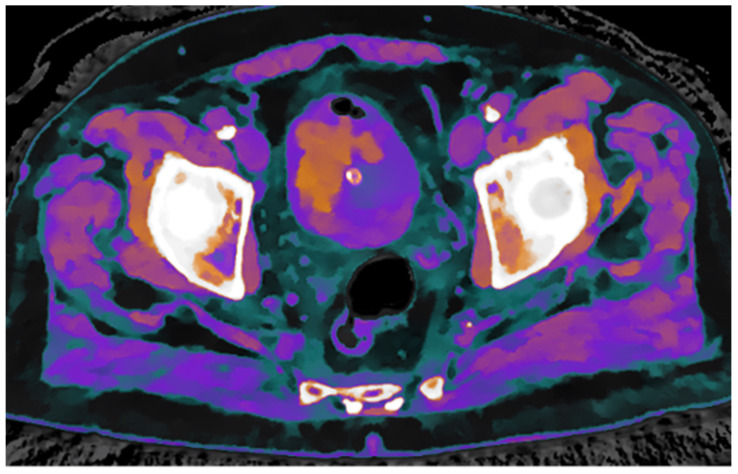
Fusion of Z-effective and electron density maps from the non-enhanced scan demonstrates an endoluminal thrombus with its characteristic structural features.

To further confirm the differing structural composition of the two tissue types, additional spectral maps were utilized. Specifically, the Z-effective map, based on the effective atomic number, combined with the electron density map, reflecting tissue electron composition, enabled a clear distinction between thrombotic and neoplastic tissue based on their unique physical properties (Figure 4).

## 3. Discussion

Accurate characterization of bladder masses is essential for precise staging and treatment planning. Although conventional multiphasic CT provides valuable anatomical detail and helps to define the extent of the lesion, intraluminal thrombus may mimic or obscure underlying neoplastic tissue, limiting accurate staging. In the present case, the use of advanced spectral CT techniques, in addition to conventional CT imaging, enabled a more reliable differentiation between bladder neoplasm and thrombus by integrating morphological assessment with iodine quantification and tissue composition analysis.

Spectral CT improves tissue characterization by differentiating materials based on their distinct responses to photoelectric absorption and Compton scattering. This approach allows for the identification of tissues by comparing their attenuation profiles to reference materials such as iodine, water, and calcium. Material decomposition algorithms enable the generation of various parametric maps, including iodine density, effective atomic number (Z-effective), and electron density maps [7].

Iodine density maps are based on the concentration of iodine expressed in mg/mL, enabling the quantification of contrast uptake. This is particularly valuable for differentiating hypervascular areas, typical of neoplastic tissue, from regions with low or absent iodine uptake, which may correspond to thrombi, necrotic tissue, or cystic formations.

The complementary information provided by both Z-effective and electron density maps offers additional insights for tissue characterization by highlighting differences in atomic number and electron density, respectively. These tools may further enhance diagnostic confidence and improve lesion differentiation [10].

In this case, relying solely on conventional imaging from the enhanced and venous phases could have led to overstaging the size of the bladder neoplasm, as the intraluminal hyperdense area appeared contiguous along both the anterior and posterior bladder walls. However, integrating conventional imaging with spectral-based maps, including iodine density, Z-effective, and electron density maps, enabled more accurate differentiation between the hypervascular neoplastic tissue and the adjacent thrombus. The additional intrinsic information provided by spectral CT technology may also aid in tumor grading. Chen et al., in a study involving 64 patients, demonstrated that spectral-derived parameters have excellent diagnostic value in differentiating between high- and low-grade bladder cancer, thus confirming the relevance and potential of this technique [10].

Accurate staging of bladder cancer is essential, as local tumor extent directly influences therapeutic strategies and patient prognosis. MRI has been increasingly adopted for local staging due to its excellent soft tissue contrast and the development of standardized protocols such as Vescical Imaging-Reporting and Data System (VI-RADS) [11]. However, MRI has limitations, including longer acquisition times, susceptibility to motion artifacts, contraindications in patients with certain implants, and limited capacity for precise tissue composition quantification.

PET-CT can offer valuable information in the assessment of distant metastatic disease, but its role in the local evaluation of bladder tumors is limited. Radiotracer excretion through the urinary tract often limits accurate characterization of intravesical lesions, and its availability, cost, and need for radiopharmaceuticals may further limit its use in routine settings [12].

On the other hand, second-generation dual-layer spectral CT enables simultaneous acquisition of high-resolution anatomical images and spectral data, without altering the acquisition protocol. Spectral maps can be generated retrospectively and contribute meaningful insights into lesion vascularity and structural composition. This allows for accurate distinction between enhancing neoplastic tissue and non-enhancing components like thrombus, which may be equivocal on conventional CT or MRI. Spectral CT thus represents a valuable complement or alternative in cases where rapid, accessible, and composition-sensitive imaging is required for clinical decision-making.

Several DECT technologies are currently available, each based on different technical principles with specific implications for clinical practice. While this case report highlights the advantages of Dual-Layer Spectral CT, Table 1 summarizes its main strengths and limitations in terms of technical performance and cost-effectiveness compared with other DECT systems.

This case report has several limitations. Spectral analysis was performed on a single CT scanner, without comparison across vendors, and the lack of standardized thresholds for spectral parameters limits reproducibility. As both the tumor and thrombus were relatively large, assessment and differentiation were facilitated; evaluating smaller, low-grade lesions would be more challenging. Finally, a longer follow-up is needed to clarify the prognostic value of these findings.

## 4. Conclusions

This case illustrates how the use of second-generation dual-layer detector-based spectral CT can significantly enhance diagnostic accuracy in the evaluation of complex bladder lesions.

Dual-energy technology has existed for years, but its application in routine clinical practice remains limited. This is primarily because many dual-energy systems require specific adjustments and protocol modifications prior to image acquisition. These additional steps can disrupt the already busy and time-constrained clinical workflow, often resulting in radiologists not taking advantage of this valuable feature. Compared to other dual-energy technologies, dual-layer spectral CT consistently acquires spectral information with every scan without requiring protocol adjustments or significant workflow changes, thereby ensuring additional data are always available to support CT image interpretation.

The numerous dual-energy–based maps generated through post-processing hold significant clinical potential, but further evidence is needed to enable their broader, more reliable, and standardized use in clinical practice.

Prospective multicenter studies are warranted to validate spectral parameters across tumor grades and histological subtypes, and to establish standardized map-specific thresholds that improve reproducibility and facilitate clinical integration. Further investigations should also assess the role of Spectral CT in treatment monitoring and in differentiating post-therapeutic changes from residual disease. Comparative studies with MRI, PET/CT, and emerging modalities such as photon-counting CT will be crucial to define the long-term role of Spectral CT in the diagnostic pathway of bladder cancer.

## Figures and Tables

**Table 1:** Main characteristics, advantages, and limitations of the spectral technologies currently available on the market.

*DECT Technology*	*Key Principles*	*Advantages*	*Limitations*
***Dual-Layer Detector***	Two scintillator layers simultaneously detect low- and high-energy photons in a single acquisition	- Always-on spectral data without protocol selection. - No need for dual scans or tube switching - Retrospective generation of spectral maps. - Minimal workflow impact	- Lower energy separation compared with source-based DECT. - Requires post-processing expertise
***Dual-Source CT***	Two X-ray tubes at different kVp levels acquire data simultaneously	- High temporal resolution - Wide energy separation improves material differentiation	- Higher cost and complexity. - Potential for cross-scatter artifacts. - Increased radiation dose in some protocols
***Rapid kVp switching (Single Source)***	One X-ray tube alternates rapidly between high and low kVp during a single rotation	- High spectral separation - Good temporal registration	- Limited to specific vendors -Requires very fast switching hardware. - May increase image noise
***Sequential*** ***(Dual Scan)***	Two separate scans at different kVp levels	- Technically simple - Widely available on older systems	- Misregistration artifacts due to motion –Increased dose - Workflow disruption

## Data Availability

The original data presented in the study are included in the article, further inquiries can be directed to the corresponding author.

## Abbreviations

The following abbreviations are used in this manuscript:

DECT	Dual-energy CT
